# Selection and Validation of Candidate Reference Genes for qRT-PCR Analysis in *Fopius arisanus*

**DOI:** 10.3390/insects17090927

**Published:** 2026-09-04

**Authors:** Muyi Chen, Xue Li, Kaixuan Ren, Cirenjiba, Xinhai Ye, Yaoyao Chen

**Affiliations:** 1 Laboratory of Insect EvoGenomics & Intelligent Computing, College of Advanced Agriculture Science (College of Plant Protection), Zhejiang A&F University, Hangzhou 311300, China; 2 Zhejiang Key Laboratory of Biology and Ecological Regulation of Crop Pathogens and Insects, Zhejiang A&F University, Hangzhou 311300, China

**Keywords:** *Fopius arisanus*, parasitoid wasp, reference genes, qRT-PCR

## Abstract

*Fopius arisanus* (Hymenoptera: Braconidae) is an important parasitoid species in tephritid fruit fly biological control and host–parasitoid interaction research. Quantitative real-time PCR (qRT-PCR) is widely used to analyze gene expression, which requires stable reference genes for accurate normalization. However, no systematic evaluation of suitable reference genes for qRT-PCR analysis across *F. arisanus* tissues has been conducted. We evaluated the expression stability of ten candidate reference genes (*Actin*, *α-tubulin*, *β-tubulin*, *RPL37a*, *EF1-α*, *EF1-δ*, *RPL4*, *RPS18*, *RPL13*, and *GAPDH*) across five adult tissues from females and males. The stability of these genes was analyzed using the ΔCt method, geNorm, NormFinder, BestKeeper, and RefFinder, and the optimal number of reference genes was determined by geNorm pairwise variation analysis. The results revealed that the optimal reference genes differed between the female and male tissue panels: *Actin* and *RPL13* were the most stable combination for female tissues, whereas *RPL13* and *RPS18* were recommended for male tissues. Notably, *β-tubulin* and *GAPDH* showed the poorest stability in female tissues, whereas *α-tubulin* and *β-tubulin* were the least stable in male tissues. The suitability of the stable and unstable reference genes was further validated by normalizing the expression of the target genes *vitellogenin* (*Vg*) and *dual-specificity phosphatase CDC14A* (*CDC14A*). Our findings represent a systematic evaluation of reference genes for *F. arisanus* and establish a reliable normalization strategy for future gene expression studies in this parasitoid wasp.

## Simple Summary

*Fopius arisanus* is a parasitoid wasp widely used to control destructive *Bactrocera* pests. Quantitative real-time PCR (qRT-PCR) is widely used to quantify gene expression. However, accurate measurement in such studies relies on the careful selection of reference genes. In this study, we assessed ten candidate genes across different tissues of male and female *F. arisanus* to identify the most reliable ones. We found that the optimal reference genes varied between the female and male tissue panels, and that using unstable genes produced misleading results. This work provides a useful basis for future molecular studies in this species.

## 1. Introduction

Quantitative real-time PCR (qRT-PCR) is one of the most widely used methods for analyzing gene expression because of its high sensitivity and specificity [1]. It has been extensively applied in insect studies to examine gene expression patterns across developmental stages, tissues, sexes, and various experimental conditions [2,3]. However, the reliability of qRT-PCR results depends largely on the selection of appropriate reference genes for normalization. Internal reference genes are expected to show stable expression across different biological samples and are commonly used to normalize qRT-PCR data. Traditional housekeeping genes, such as *Actin*, *tubulin*, *glyceraldehyde-3-phosphate dehydrogenase* (*GAPDH*), *elongation factor-1 alpha* (*EF1-α*), and *ribosomal protein genes*, have been widely used as internal controls [2,4,5]. Nevertheless, increasing evidence has shown that the expression of these genes is not always constant, and their stability may vary among species, tissues, developmental stages, sexes, and experimental treatments [5,6]. The use of unstable reference genes can lead to inaccurate normalization and may distort the apparent expression patterns of target genes [6,7]. Therefore, systematic validation of candidate reference genes is essential for reliable qRT-PCR-based gene expression analysis.

*Fopius arisanus* (Hymenoptera: Braconidae) is an egg-pupal endoparasitoid of tephritid fruit flies and an important biological control agent against economically important *Bactrocera* pests [8]. It has been successfully established in Hawaii and French Polynesia, where it contributed to the suppression of *Bactrocera dorsalis* and other fruit fly populations, and has been widely studied in classical biological control, host suitability, and tri-trophic interactions [9,10,11]. Recent genomic and transcriptomic advances in *F. arisanus* have yielded valuable molecular resources, including a whole-genome assembly [12], transcriptomic datasets across developmental stages [13], and *endogenous nudivirus* (*FaENV*) genes associated with virus-like particle production in the female reproductive system [14]. These advances provide candidate genes for understanding the molecular basis of development, reproduction, and host–parasitoid interactions in *F. arisanus*. However, to translate these resources into functional understanding, accurate gene expression quantification is essential. To date, reference genes for qRT-PCR normalization in *F. arisanus* have not been systematically validated across different tissues, representing a critical gap for reliable gene expression analysis in *F. arisanus*.

In the present study, we evaluated the expression stability of ten candidate reference genes, including *Actin*, *alpha-tubulin* (*α-tubulin*), *beta-tubulin* (*β-tubulin*), *ribosomal protein L37a* (*RPL37a*), *EF1-α*, *elongation factor 1-delta* (*EF1-δ*), *ribosomal protein L4* (*RPL4*), *ribosomal protein S18* (*RPS18*), *ribosomal protein L13* (*RPL13*), and *GAPDH* in *F. arisanus* across different male and female tissues. Reference gene stability was evaluated by ΔCt, geNorm, NormFinder, BestKeeper, and RefFinder, and the optimal gene number was determined by geNorm pairwise variation (Vn/Vn + 1) analysis. Finally, selected stable and unstable reference genes were used to normalize the expression of target genes to validate the impact of reference gene selection on qRT-PCR results. This study provides a systematic evaluation of reference genes for *F. arisanus* and establishes a reliable normalization strategy for future gene expression studies in this important biological control parasitoid.

## 2. Materials and Methods

### 2.1. Insect Rearing and Sample Preparation

*Fopius arisanus* was maintained in an insectary at 24 ± 2 °C, 55 ± 10% relative humidity, and a photoperiod of 14 h:10 h (L:D). Adult wasps were provided with a 20% (*w*/*v*) honey solution [15]. Seven-day-old male and female adults were dissected in sterile phosphate-buffered saline (PBS) under a stereomicroscope (SMZ161; Motic, Xiamen, China) for tissue collection. Individual wasps were dissected sequentially in ice-cold sterile PBS, and tissues were collected from individual wasps one by one before being pooled to generate each biological replicate. To minimize potential cross-contamination between tissue types, the dissection forceps were thoroughly cleaned with 75% ethanol before dissecting each new tissue type. For females, the head, fat body, gut, ovaries, and venom gland were collected; for males, the head, fat body, gut, testes, and Hagen’s gland (a male-specific gland on the internal surface of the 9th abdominal tergum) were collected. Tissues shared by both sexes (head, fat body, and gut) were collected separately for females and males without pooling across sexes. The dissected tissues were immediately transferred into RNase-free microcentrifuge tubes, frozen in liquid nitrogen, and stored at −80 °C until total RNA extraction. Each biological replicate consisted of an independent pool of tissues from 20 individual wasps; each tissue type had three independent biological replicates (60 wasps in total per tissue type).

### 2.2. RNA Extraction and cDNA Synthesis

Total RNA was extracted from each sample using FreeZol Reagent (R711; Vazyme, Nanjing, China) according to the manufacturer’s instructions. RNA purity and concentration were quantified via spectrophotometer (Eva 3200, Monad, Suzhou, China), and samples with A260/A280 ratios between 1.8 and 2.0 were used for subsequent analyses. Genomic DNA was removed and first-strand cDNA was synthesized from 50 ng of total RNA using HiScript III RT SuperMix for qPCR (+gDNA wiper) (R323-01; Vazyme, China). The final volume of each reverse-transcription reaction was 20 μL according to the manufacturer’s protocol. After synthesis, the cDNA was diluted 1:7 (*v*/*v*) with nuclease-free water and stored at −20 °C until use.

### 2.3. Primer Design and Standard Curve Construction

Fragments of ten candidate reference genes (*Actin*, *α-tubulin*, *β-tubulin*, *RPL37a*, *EF1-α*, *EF1-δ*, *RPL4*, *RPS18*, *RPL13*, and *GAPDH*) and target genes *vitellogenin* (*Vg*) and *dual-specificity phosphatase CDC14A* (*CDC14A*) were amplified from cDNA. The purified PCR products were separately cloned into the pClone007 Blunt Vector (CV16; Vazyme, China). The recombinant vectors were then transformed into DH5α *Escherichia coli* competent cells (Tsingke, Beijing, China). Positive recombinant plasmids were extracted using a plasmid extraction kit (DP103, TIANGEN, Beijing, China). For each gene, plasmids were serially diluted 10-fold to generate six to eight dilution points for standard curve construction. Cq values were plotted against the logarithm of plasmid copy numbers, and the correlation coefficient (R^2^) was calculated from the linear regression equation. The amplification efficiency was calculated using the equation Efficiency (%) = [10^(−1/slope) − 1] × 100 [16,17]. Primer sequences, product lengths, amplification efficiencies, R^2^ values, and slopes of the candidate reference and target genes are listed in Table 1.

### 2.4. Quantitative Real-Time PCR (qRT-PCR) Analysis

qRT-PCR was performed on a LightCycler 480 II Real-Time PCR System (Roche, Basel, Switzerland) using ChamQ SYBR Color qPCR Master Mix (Q411-02, Vazyme, China). Each 10 μL reaction contained 5 μL of 2× master mix, 0.2 μL of each primer (10 μM), 1 μL of cDNA template, and 3.6 μL of nuclease-free water. The cycling program consisted of an initial denaturation at 95 °C for 30 s, followed by 40 cycles of 95 °C for 10 s and 60 °C for 30 s. Three independent biological replicates were analyzed, each with three technical replicates.

### 2.5. Determination of Reference Gene Expression Stability

The expression stability of the ten candidate reference genes was evaluated using the comparative ΔCt method [18], BestKeeper [19], NormFinder, and geNorm [6]. The comprehensive ranking of candidate reference genes was obtained using RefFinder (https://blooge.cn/RefFinder/, v2020.10.20). The optimal number of reference genes required for normalization was determined by geNorm based on pairwise variation (Vn/Vn + 1). A threshold value of 0.15 was applied, and when Vn/Vn + 1 was below 0.15, n reference genes were considered sufficient for reliable normalization.

### 2.6. Validation of Reference Genes

To further evaluate the suitability of the selected reference genes, *Vg* and *CDC14A* were used as target genes to assess the performance of the stable reference genes *RPL13* and *RPS18*, individually and in combination, and the unstable reference gene *GAPDH*. The expression levels of the target genes were analyzed across different tissues of male and female adults. Relative mRNA expression levels were calculated using the 2^−ΔΔCt^ method. Statistical analysis was performed using one-way analysis of variance (ANOVA) followed by Tukey’s multiple comparisons test in GraphPad Prism 8.0.

## 3. Results

### 3.1. Primer Specificity and Amplification Efficiency

Prior to evaluating the expression stability of candidate reference genes, the specificity and amplification efficiency of the ten candidate reference gene primer pairs were assessed. Agarose gel electrophoresis showed that each candidate reference gene primer pair amplified a single band of the expected size, ranging from 89 to 193 bp (Figure S1). Standard curves were generated using 10-fold serial dilutions of recombinant plasmids to determine the amplification efficiency and linear correlation of each primer pair. The amplification efficiencies of the ten candidate reference gene primer pairs ranged from 90.5% to 106.9%, and the correlation coefficients ranged from 0.9908 to 1.0000 (Table 1; Figure S2). The slopes of the standard curves ranged from −3.573 to −3.167, indicating good amplification performance. These results demonstrated that all ten candidate reference gene primer pairs showed high specificity and acceptable amplification efficiency, and were therefore suitable for subsequent reference gene stability analysis.

### 3.2. Expression Profiles of Candidate Reference Genes

**Figure 1:**
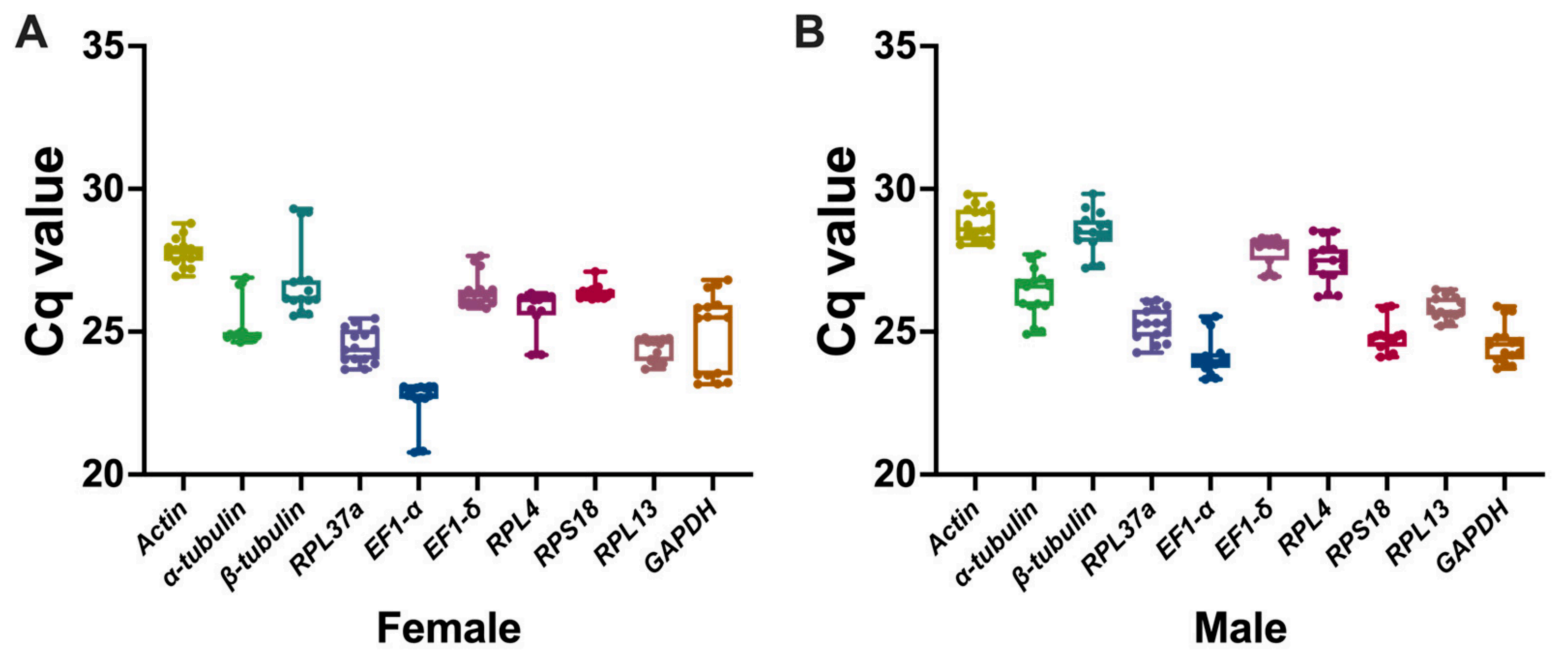
Expression profiles of ten candidate reference genes in different tissues of female (**A**) and male (**B**) *F. arisanus*. Boxes indicate the interquartile range, horizontal lines represent medians, and whiskers show minimum and maximum values.

The Cq values of the ten candidate reference genes were analyzed to evaluate their expression levels and variation across different tissues of female and male *F. arisanus*. The Cq distribution showed that the candidate reference genes exhibited different expression levels among tissues (Figure 1). Among these candidates, *EF1-α* and *Actin* exhibited the highest and lowest expression levels, respectively, across both sexes. Based on the Cq distribution, *RPL13* showed relatively small variation across tissues within each sex, approximately 0.9 cycles in the female tissue panel and approximately 0.8 cycles in the male tissue panel, whereas *GAPDH*, *α-tubulin*, and *β-tubulin* exhibited pronounced tissue-dependent fluctuations, with variation (range) exceeding 2 cycles in the female tissue panel and approximately 2–3 cycles in the male tissue panel (Figure 1). These results indicated that the expression levels of candidate reference genes varied among tissues, highlighting the need for systematic stability evaluation before target gene normalization.

### 3.3. Stability Analysis of Candidate Reference Genes in Female and Male Tissues

To systematically evaluate the stability of the ten candidate reference genes in different tissues of female and male *F. arisanus*, four commonly used algorithms, including the comparative ΔCt method, NormFinder, BestKeeper, and geNorm, were applied. Because different algorithms may generate different rankings, RefFinder was further used to integrate the results and provide a comprehensive ranking of candidate reference genes.

**Figure 2:**
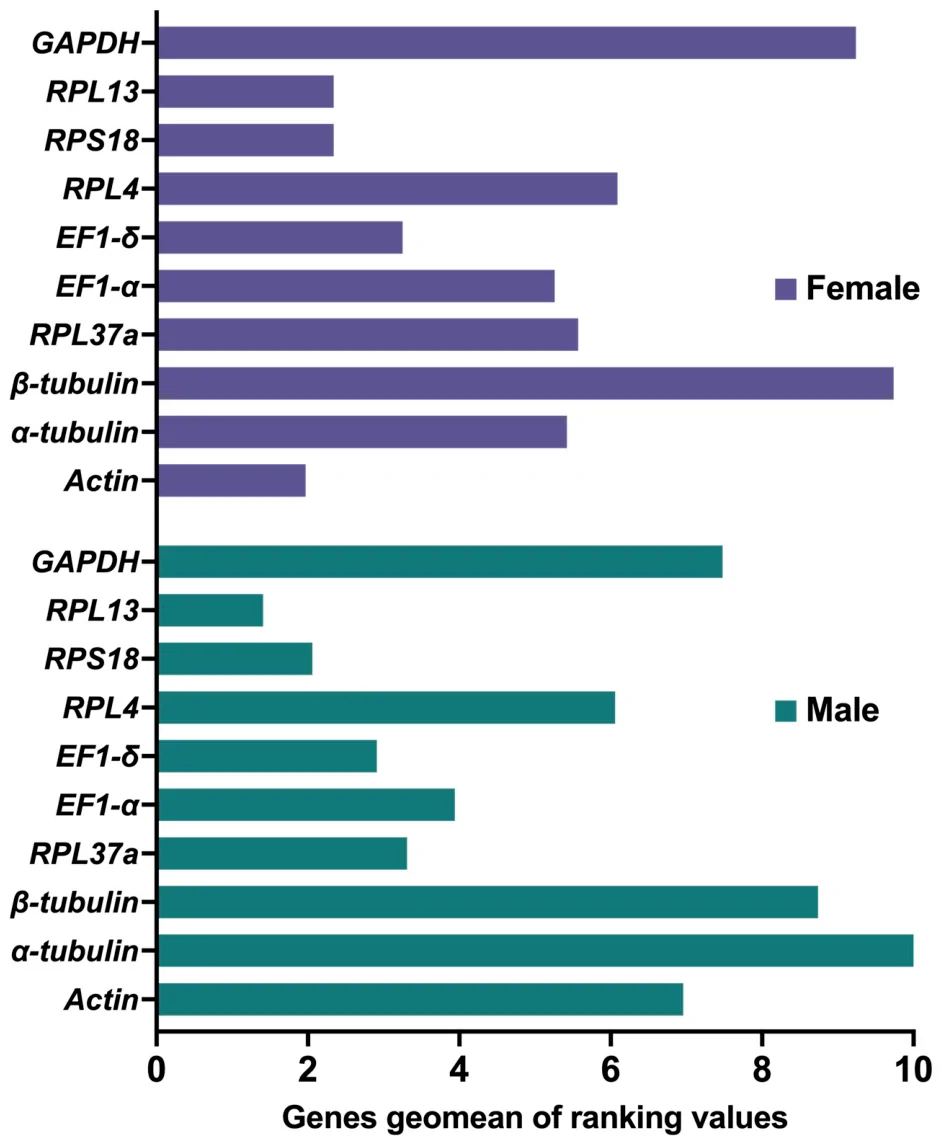
Comprehensive ranking of candidate reference genes in female and male *F. arisanus* based on RefFinder analysis. The geometric mean value was used to indicate the overall expression stability of each candidate reference gene. Lower geometric mean values indicate higher expression stability.

**Figure 3:**
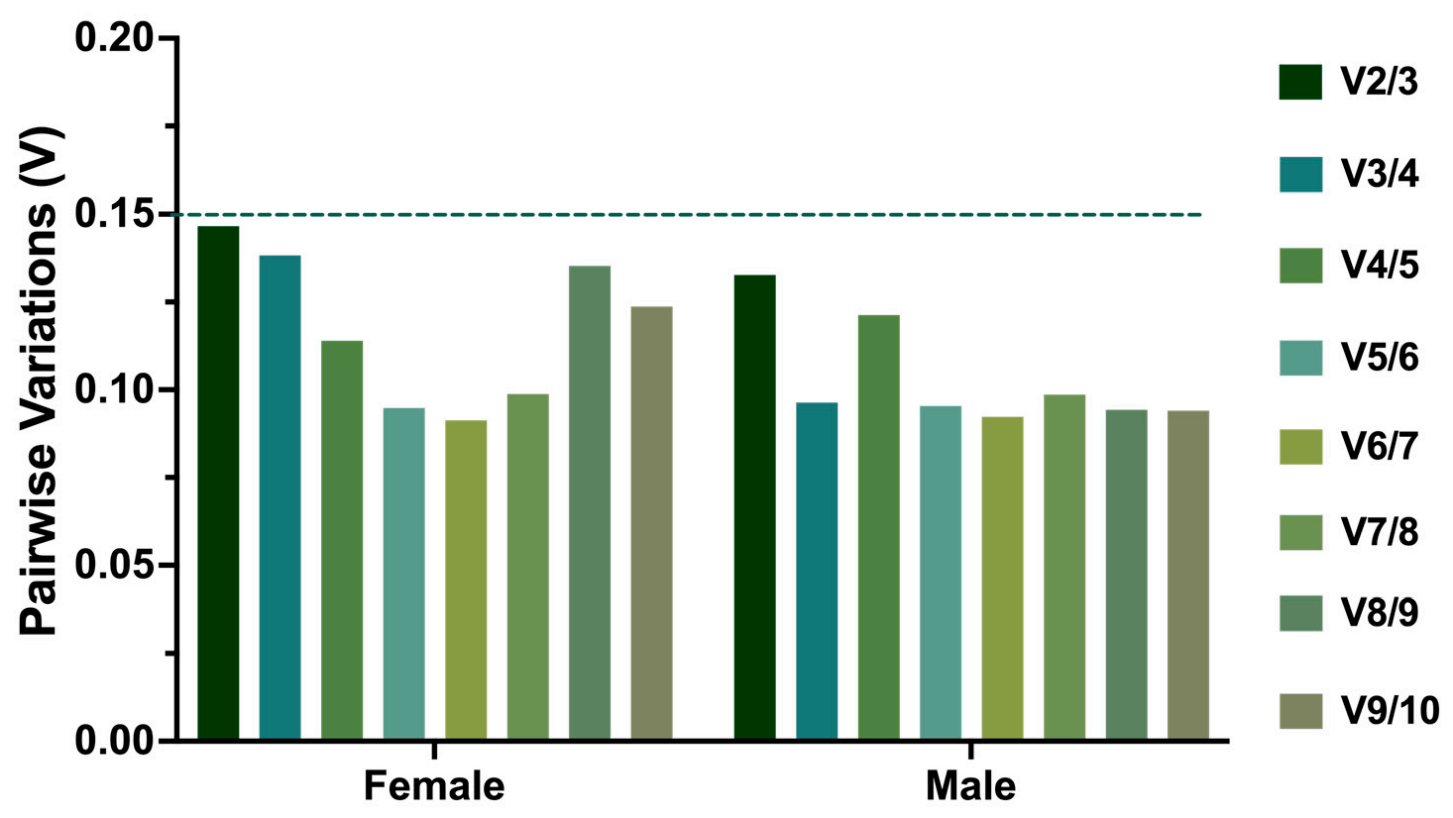
Determination of the optimal number of reference genes for normalization in *F. arisanus*. Pairwise variation (Vn/Vn + 1) values were calculated using geNorm. The dashed line indicates the threshold value of 0.15; values below this threshold suggest that n reference genes are sufficient for reliable normalization.

For female tissues, the comparative ΔCt method and NormFinder both identified *Actin* as the most stable candidate reference gene, followed by *EF1-δ* and *RPL13* (Table 2). BestKeeper and geNorm identified *RPL13* and *RPS18* as the two most stable genes, which yielded the lowest SD and M-values, respectively (Table 2). Although the rankings differed slightly among the four algorithms, *Actin*, *RPL13*, and *RPS18* consistently showed relatively high stability in female tissues. By contrast, *β-tubulin* and *GAPDH* were ranked among the least stable genes by most algorithms, occupying the last or second-last positions in all four analyses (Table 2), indicating that they were not suitable for normalization in female tissue samples. According to the comprehensive ranking generated by RefFinder, the overall stability of the candidate reference genes in female tissues was as follows: *Actin* > *RPL13* > *RPS18* > *EF1-δ* > *EF1-α* > *α-tubulin* > *RPL37a* > *RPL4* > *GAPDH* > *β-tubulin* (Figure 2). Furthermore, geNorm pairwise variation analysis showed that the V2/3 value was 0.1466, which was below the recommended threshold of 0.15 (Figure 3). This result indicated that two reference genes were sufficient for accurate normalization in female tissues. Therefore, *Actin* and *RPL13* were recommended as the optimal reference gene combination for female tissue samples.

For male tissues, *RPL13* showed consistently high stability, ranking first in the comparative ΔCt method, NormFinder, and BestKeeper analyses. *EF1-δ* and *RPS18* also showed relatively high stability, and *RPS18* was ranked the most stable gene by geNorm. By contrast, *α-tubulin* and *β-tubulin* exhibited the poorest stability performance, occupying the bottom two ranks across all four algorithms. Thus, they are not recommended as reference genes for normalization in male tissues. According to RefFinder comprehensive analysis, the overall stability ranking of the candidate reference genes in male tissues was as follows: *RPL13* > *RPS18* > *EF1-δ* > *RPL37a* > *EF1-α* > *RPL4* > *Actin* > *GAPDH* > *β-tubulin* > *α-tubulin* (Figure 2). GeNorm pairwise-variation analysis indicated that two reference genes were adequate for male-tissue samples. Thus, *RPL13* and *RPS18* were identified as the optimal reference gene combination for male tissue samples.

### 3.4. Validation of Candidate Reference Genes

To validate the selected reference genes, the expression levels of *dual-specificity phosphatase CDC14A* (*CDC14A*) and *Vitellogenin* (*Vg*) were analyzed in female and male tissue panels. The primer specificities for *CDC14A* and *Vg* were verified by agarose gel electrophoresis, yielding single amplification products of the expected fragment sizes (141 bp and 191 bp, respectively) (Figure S3). Standard-curve analysis showed that the amplification efficiencies of *CDC14A* and *Vg* were 91.1% and 103.4%, respectively, comparable to those of the validated reference genes (90.5–106.9%), thus satisfying the assumption underlying the 2^−ΔΔCt^ relative quantification method (Table 1; Figure S4). The target genes were normalized using the stable reference genes *RPS18*, *RPL13*, and *RPL13/RPS18*, as well as the unstable reference gene *GAPDH*. For consistency in the validation analyses, *RPL13/RPS18* was used for normalization in both the female and male tissue panels.

**Figure 4:**
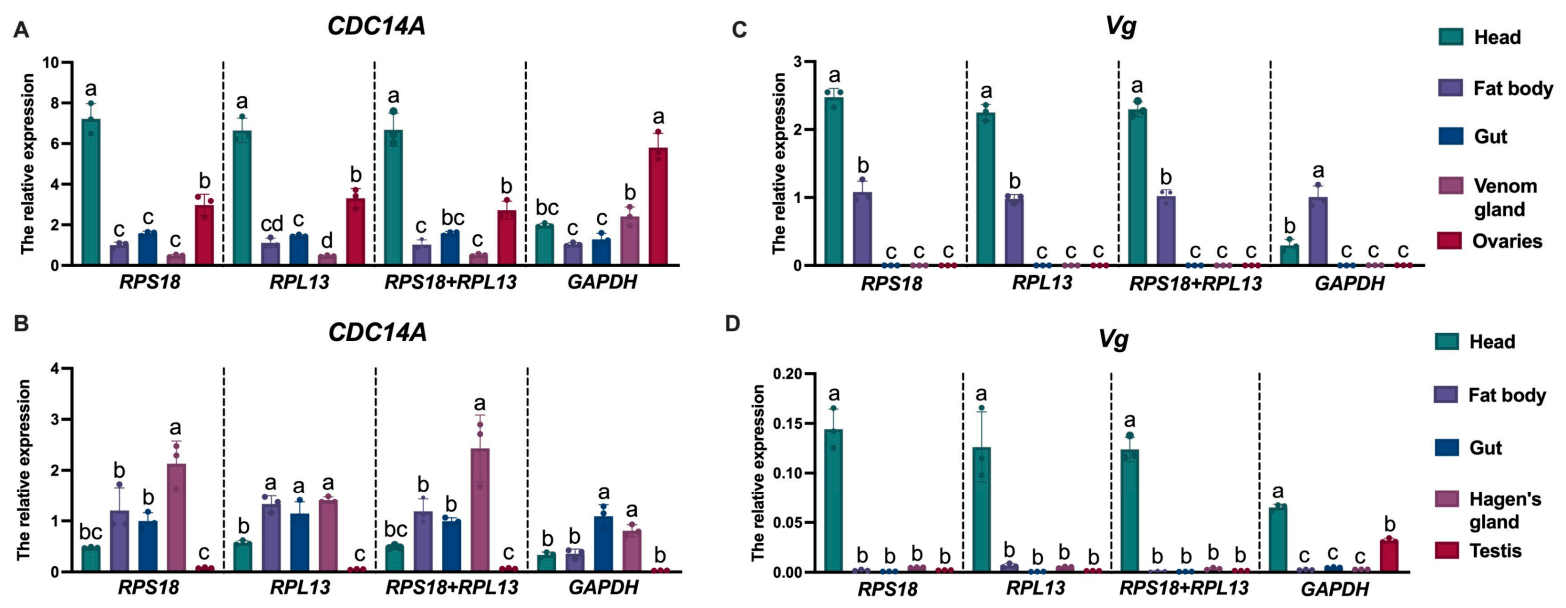
Validation of selected reference genes using target gene expression analysis in female and male *F. arisanus*. Relative expression levels of *Vg* and *CDC14A* were normalized using the stable reference genes *RPS18*, *RPL13*, and *RPL13/RPS18*, as well as the unstable reference gene *GAPDH*. (**A**,**C**): expression patterns of *CDC14A* and *Vg* in female tissues; (**B**,**D**): expression patterns of *CDC14A* and *Vg* in male tissues. Data are shown as mean ± SE from three biological replicates. Different letters indicate significant differences among tissues based on one-way ANOVA followed by Tukey’s multiple comparison test (*p* < 0.05).

For *CDC14A*, normalization with *RPS18*, *RPL13*, or *RPL13/RPS18* produced similar expression patterns. In female tissues, *CDC14A* showed the highest expression in the head, followed by the ovaries (Figure 4A). In male tissues, *CDC14A* was relatively highly expressed in the Hagen’s gland and showed the lowest expression in the testis. However, when *GAPDH* was used for normalization, the expression pattern was altered, particularly in female tissues, where *CDC14A* was no longer most highly expressed in the head but instead showed the highest expression in the ovaries. A similar discrepancy was also observed for *Vg* expression. For *Vg*, normalization with the stable reference genes showed that *Vg* was mainly expressed in the female head and fat body, while its expression in the gut, venom gland, and ovary was extremely low. In male tissues, *Vg* expression was generally low, with relatively higher expression detected in the head. In contrast, normalization with *GAPDH* changed the apparent expression pattern.

Overall, these results confirmed that *RPL13* and *RPS18* are reliable reference genes for expression analysis across female and male tissues in *F. arisanus*. The use of *GAPDH* led to altered expression profiles, indicating that unstable reference genes may cause biased interpretation of target gene expression.

## 4. Discussion

The present study showed that candidate reference gene stability differed markedly between female and male tissues of *F. arisanus*. This variation between tissue panels is consistent with previous reports in other insect orders, including Hemiptera, Lepidoptera, Coleoptera, and Diptera, in which optimal reference genes differed among species, tissues, developmental stages, and experimental treatments [2,5,20,21,22]. These findings reinforce that commonly used housekeeping genes cannot be assumed to be universally stable, and highlight the need for empirical validation before reference genes are adopted for qRT-PCR normalization.

Among the tested candidate genes, *GAPDH* was not recommended as a suitable reference gene for either female or male tissues of *F. arisanus*. Although *GAPDH* was identified as a suitable reference gene for tissue samples in some insects, such as *Bombyx mori* [23] and *Spodoptera exigua* [24], it showed poor stability in larval tissues of *Chlorops oryzae* [25]. The poor stability of *GAPDH* in *F. arisanus* may be related to its biological functions, as *GAPDH* encodes a key glycolytic enzyme involved in energy metabolism, development, and hormone-regulated processes [26]. In addition, *GAPDH* homologs have been detected in venom gland or venom-associated transcriptomes or proteomic studies of several parasitoid wasps, including *Leptopilina*, *Ganaspis*, and *Psyttalia* species [27,28,29,30]. In the present study, *GAPDH* showed pronounced tissue-dependent variation, particularly in female tissues, suggesting that its expression may be influenced by tissue function or physiological state in *F. arisanus*. Although the presence of *GAPDH* in parasitoid venom-associated studies raises the possibility that it may be associated with venom gland physiology [29,30], further evidence is required to determine whether *GAPDH* has a venom-related role in *F. arisanus*. Regardless of its specific function, its marked tissue-dependent variation makes *GAPDH* unsuitable as a reference gene for cross-tissue normalization. Similarly, *α-tubulin* and *β-tubulin* also showed poor stability, suggesting that cytoskeleton-related housekeeping genes are not necessarily suitable internal controls for tissue-specific expression analysis in *F. arisanus*.

In contrast, *ribosomal protein genes* are frequently identified as relatively stable reference genes in insects [31]. For example, *RPL13* and *RPS18* have been recommended in *Aphidius gifuensis* for developmental, tissue, or sex-related comparisons [32], and both genes also ranked among the stable candidates in *Lipaphis erysimi* [33]. Consistent with these studies, *RPL13* exhibited high stability in both female and male tissues of *F. arisanus*, ranking as the most stable gene in males and among the top two in females. *RPS18* also showed high stability, particularly in male tissues where it ranked second. Their relatively stable expression may be associated with their roles in ribosome structure and basal protein synthesis [34,35]. Notably, *Actin* was identified as the most stable gene in female tissues and was therefore included in the optimal combination with *RPL13*. However, its stability ranking dropped substantially in male tissues, further highlighting the tissue panel-specific nature of reference gene performance. Nevertheless, reference genes should still be empirically validated according to the target species, tissue types, and experimental conditions [36,37].

The target-gene validation further demonstrated the practical impact of reference gene selection on expression analysis. Based on the stability analyses, *Actin/RPL13* was identified as the optimal combination for the female tissue panel, whereas *RPL13*/*RPS18* was identified as the optimal combination for the male tissue panel. The use of the same reference-gene combination can reduce potential normalization bias caused by different internal control systems between tissues. Normalization with the stable reference genes produced generally consistent expression patterns, whereas the unstable reference gene *GAPDH* altered the apparent expression profiles of both target genes, confirming that inappropriate reference genes can lead to biased interpretation of gene expression [3,38,39]. *CDC14A* encodes a dual-specificity protein phosphatase and may be associated with cell-cycle regulation or tissue-specific physiological activity in *F. arisanus* [40]. For *Vg*, relatively high expression was detected in the female head and fat body. *Vg* is traditionally considered to be mainly synthesized in the fat body and transported to the ovary for yolk deposition and oocyte maturation [41]. However, the relatively high expression of *Vg* in the head may reflect a tissue-specific role beyond classical yolk protein synthesis, though this interpretation requires further validation [42,43]. Overall, these results demonstrate that the selection of reference genes can directly affect the biological interpretation of target-gene expression and highlight the importance of validated normalization systems for future functional studies in *F. arisanus*.

## 5. Conclusions

This study evaluated ten candidate reference genes in female and male tissues of *Fopius arisanus*. The optimal reference genes differed between the female and male tissues: *Actin/RPL13* was recommended for female tissues, whereas *RPL13/RPS18* was recommended for male tissues. *GAPDH* and *tubulin* showed poor stability and are not suitable for tissue-specific normalization in the respective tissue panels. Validation with *CDC14A* and *Vg* further demonstrated that reference-gene selection can substantially affect qRT-PCR expression profiles in *F. arisanus*.

## Figures and Tables

**Table 1:** Primer information for candidate reference genes and target genes used for qRT-PCR in *Fopius arisanus*.

Gene Name	Accession No.	Primer Sequence	Product Length	Efficiency (%)	R^2^	Slope
Actin	XP_011307078.1	TGATAATGGAACTGGGTTTG	107	91.0	0.9952	−3.531
		TTTTGTTGACGGCTCTGATA				
β-tubulin	XP_011304849.1	TCAAGCTATCGACCCCAACC	89	103.6	0.9985	−3.238
		ACCAGGGAACCTCAGGCAAG				
α-tubulin	XP_011312142.1	GACAATGAGGCGATTTACGA	193	92.3	0.9995	−3.521
		GAGGGAAATGGATTCTTGGA				
RPL37a	XP_011304587.1	GATGAAACGCAGTGTTGTTG	135	106.9	0.9955	−3.167
		TCCTTAACTTCACGCAGTCG				
EF1-α	XP_011304460.1	TACCACCCAGCAGACCTACG	174	90.5	1.0000	−3.573
		TCTCCACGGATTTTACCTCA				
EF1-δ	XP_011309605.1	GTACAACGCCAAGAAGTCCA	146	98.5	0.9980	−3.359
		CTCCCCAGAGTAAACCATCC				
RPL4	XP_011298139.1	GTCGTGCTGTCGCTCGTATT	117	100.5	0.9985	−3.310
		CTGTATGGCTTAGTCGGTGC				
RPS18	XP_011301543.1	GAAAATACTCCCAGTTGACG	178	94.2	0.9923	−3.471
		CTTCTTCTTGGATACACCGA				
RPL13	XP_011301440.1	TGCCCGACAGTTCGCTACCA	114	95.2	0.9908	−3.444
		CCACGGAGATGCCAATGCTC				
GAPDH	XP_011305510.1	TGAAGGTGGTTGTTTAGTTG	140	96.2	0.9962	−3.416
		AGGCTTTCTCGATAGTGGTA				
CDC14A	XM_011312691.1	GCCTACGAGTTGCTGATGAA	141	91.1	0.9930	−3.554
		GAAGAAGCCGAGACGATGAC				
Vg	XM_011307394.1	GCAAGAAGCCATCCACTGTT	191	103.4	0.9966	−3.243
		CTGAAGCATCGCAAGAGGAG				

Note: Efficiency (%) = [10^(−1/slope) − 1] × 100. Efficiency, R^2^, and slope were determined for candidate reference genes as well as target genes *CDC14A* and *Vg*.

**Table 2:** Stability rankings of candidate reference genes in female and male tissues of *F. arisanus* using different algorithms.

Experiment Condition	Candidate Genes	ΔCt Method SD	Rank	NormFinder SV	Rank	BestKeeper SD	Rank	geNorm	Rank
								M	
Female	*Actin*	0.718	1	0.285	1	0.497	3	0.566	5
	*α-tubulin*	0.908	6	0.519	6	0.857	6	0.689	8
	*β-tubulin*	1.423	10	1.040	10	1.410	9	0.924	10
	*RPL37a*	0.995	8	0.617	7	0.645	5	0.423	3
	*EF1-α*	0.897	5	0.438	3	0.947	8	0.595	6
	*EF1-δ*	0.791	2	0.363	2	0.622	4	0.633	7
	*RPL4*	0.929	7	0.621	8	0.867	7	0.510	4
	*RPS18*	0.888	4	0.474	5	0.195	1	0.404	2
	*RPL13*	0.850	3	0.459	4	0.423	2	0.350	1
	*GAPDH*	1.407	9	0.973	9	1.525	10	0.823	9
Male	*Actin*	0.885	7	0.647	8	0.608	4	0.676	8
	*α-tubulin*	1.104	10	0.681	9	0.930	10	0.800	10
	*β-tubulin*	0.975	9	0.722	10	0.778	8	0.737	9
	*RPL37a*	0.764	5	0.514	6	0.602	3	0.371	2
	*EF1-α*	0.757	4	0.434	4	0.744	7	0.390	3
	*EF1-δ*	0.710	2	0.393	2	0.508	2	0.560	6
	*RPL4*	0.778	6	0.457	5	0.825	9	0.513	5
	*RPS18*	0.741	3	0.412	3	0.619	5	0.368	1
	*RPL13*	0.647	1	0.359	1	0.394	1	0.418	4
	*GAPDH*	0.915	8	0.646	7	0.743	6	0.610	7

Note: SD: Standard Deviation, SV: Stability Value, M: Average expression stability value.

## Data Availability

Data are contained within this article or the Supplementary Materials.

## Supplementary Materials

The following supporting information can be downloaded at https://www.mdpi.com/article/10.3390/insects17090927/s1, Figure S1: Specificity of PCR amplification for the ten candidate reference genes in *F. arisanus.* PCR products were separated by agarose gel electrophoresis. M, DNA marker; lanes 1–10 represent *Actin*, *α-tubulin*, *β-tubulin*, *RPL37a*, *EF1-α*, *EF1-δ*, *RPL4*, *RPS18*, *RPL13*, and *GAPDH*, respectively; Figure S2: Quantitative real-time PCR amplification curves of ten candidate reference genes in *F. arisanus*. A–J represent *Actin*, *α-tubulin*, *β-tubulin*, *RPL37a*, *EF1-α*, *EF1-δ*, *RPL4*, *RPS18*, *RPL13*, and *GAPDH*, respectively; Figure S3: Specificity of PCR amplification for the target genes *Vg* and *CDC14A* in *F. arisanus*. Amplicons were separated by agarose gel electrophoresis. M: DNA marker; lane 1: *Vg*; lane 2: *CDC14A*; Figure S4: Standard curves for the target genes *CDC14A* (A) and *Vg* (B) in *F. arisanus*. Cq values were plotted against the logarithm of plasmid copy numbers. The amplification efficiencies were 91.1% for *CDC14A* and 103.4% for *Vg*.
