# Supplementary material for: Selection and Validation of Candidate Reference Genes for qRT-PCR Analysis in Fopius arisanus
Source: Insects. 2026 Sep 4;17(9):927. doi: 10.3390/insects17090927 (PMC13607656; doi:10.3390/insects17090927)
Supplement: Supplementary file 1 [file insects-17-00927-s001.zip › insects-4516053-supplementary.pdf]

Supporting Information for

Selection and Validation of Candidate Reference Genes for qRT-PCR Analysis in *Fopius arisanus*

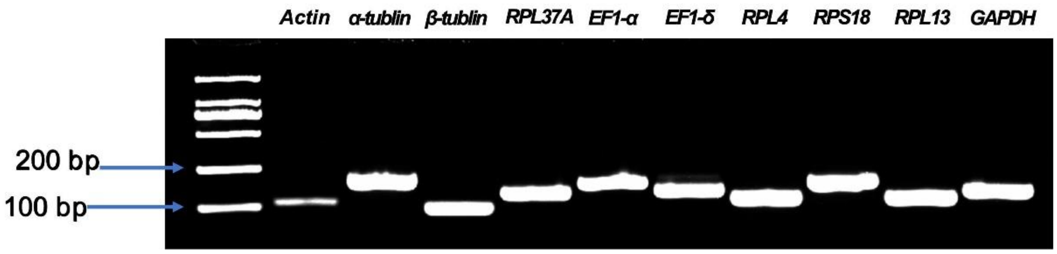

Figure S1. Specificity of PCR amplification for the ten candidate reference genes in *F. arisanus*. PCR products were separated by agarose gel electrophoresis. M, DNA marker; lanes 1–10 represent *Actin, α-tubulin, β-tubulin, RPL37a, EF1-α, EF1-δ, RPL4, RPS18, RPL13, and GAPDH*, respectively.

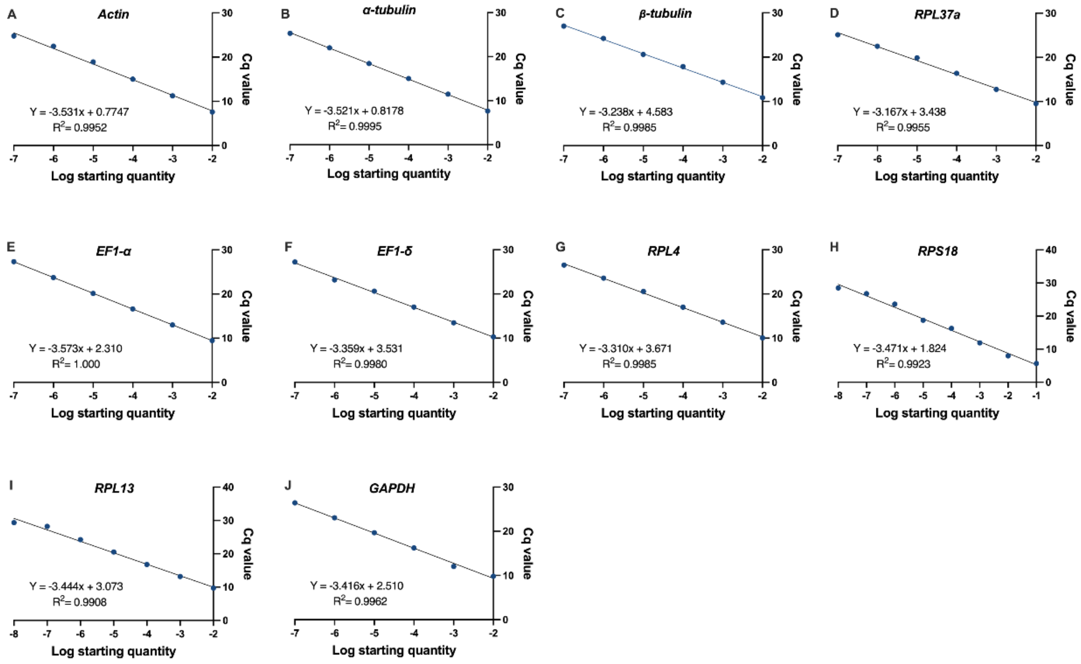

Figure S2. Quantitative real-time PCR amplification curves of ten candidate reference genes in *F. arisanus*. A–J represent *Actin, α-tubulin, β-tubulin, RPL37a, EF1-α, EF1-δ, RPL4, RPS18, RPL13, and GAPDH*, respectively.

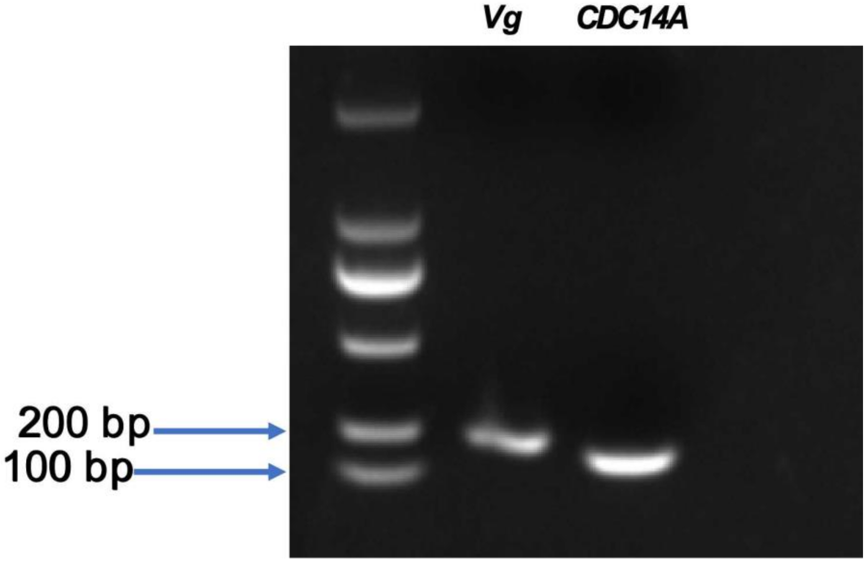

Figure S3. Specificity of PCR amplification for the target genes *Vg* and *CDC14A* in *F. arisanus*. Amplicons were separated by agarose gel electrophoresis. M: DNA marker; lane 1: *Vg*; lane 2: *CDC14A*.

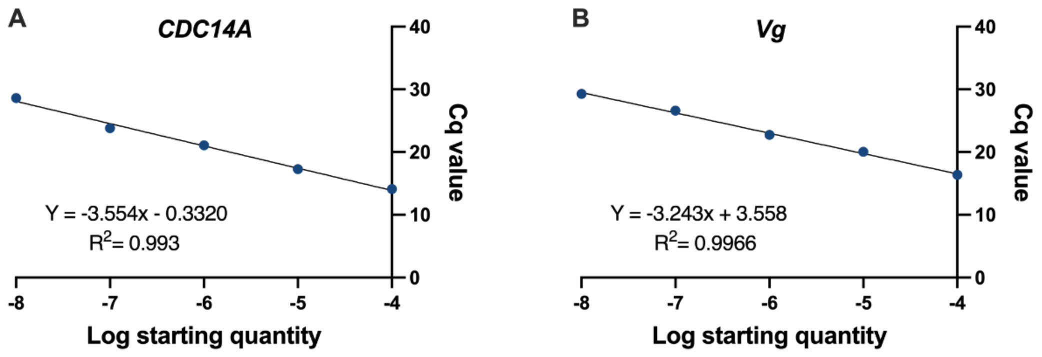

Figure S4. Standard curves for the target genes *CDC14A* (A) and *Vg* (B) in *F. arisanus*. Cq values were plotted against the logarithm of plasmid copy numbers. The amplification efficiencies were 91.1% for *CDC14A* and 103.4% for *Vg*.
